# A family with type A insulin resistance syndrome caused by a novel insulin receptor mutation

**DOI:** 10.1530/EDM-22-0362

**Published:** 2023-04-12

**Authors:** Osamu Horikawa, Satoshi Ugi, Tomofumi Takayoshi, Yasushi Omura, Maya Yonishi, Daisuke Sato, Yukihiro Fujita, Tomoya Fuke, Yushi Hirota, Wataru Ogawa, Hiroshi Maegawa

**Affiliations:** 1 Department of Medicine, Shiga University of Medical Science, Otsu, Shiga, Japan; 2 Department of Medicine, Omihachiman Community Medical Center, Omihachiman, Shiga, Japan; 3 Division of Diabetes and Endocrinology, Kobe University Graduate School of Medicine, Kobe, Japan; 4 Department of Internal Medicine, Kohka Public Hospital, Kohka, Shiga, Japan; 5 Department of Medicine, Saiseikai Shiga Hospital, Ritto, Shiga, Japan

**Keywords:** Adolescent/young adult, Male, Asian - Japanese, Japan, Pancreas, Diabetes, Unique/unexpected symptoms or presentations of a disease, April, 2023

## Abstract

**Summary:**

A 17-year-old boy was referred to our endocrinology clinic for a clinical investigation of hyperinsulinemia. An oral glucose tolerance test showed plasma glucose concentrations in the normal range. However, insulin concentrations were considerably elevated (0 min: 71 μU/mL; 60 min: 953 μU/mL), suggesting severe insulin resistance. An insulin tolerance test confirmed that he had insulin resistance. There was no apparent hormonal or metabolic cause, including obesity. The patient had no outward features of hyperinsulinemia, including acanthosis nigricans or hirsutism. However, his mother and grandfather also had hyperinsulinemia. Genetic testing showed that the patient (proband), his mother, and his grandfather had a novel p.Val1086del heterozygous mutation in exon 17 of the insulin receptor gene (*INSR*). Although all three family members have the same mutation, their clinical courses have been different. The onset of the mother’s diabetes was estimated at 50 years, whereas the grandfather developed diabetes at 77 years.

**Learning points:**

- Type A insulin resistance syndrome is caused by mutations in the insulin receptor (*INSR*) gene and results in severe insulin resistance.
- Genetic evaluation should be considered in adolescents or young adults with dysglycemia when an atypical phenotype, such as severe insulin resistance, or a relevant family history is observed.
- Clinical courses may differ even if the same genetic mutation is found in a family.

## Background

Type A insulin resistance syndrome (IRS) is caused by mutations in the insulin receptor (*INSR*) gene (1, 2). IRS is a rare disorder, but recent findings suggest that at least 0.05% of the population harbors a pathological *INSR* mutation (3). Because this disorder has a mild phenotype and is not well understood, many of these patients are misdiagnosed and incorrectly treated. We report a family with type A IRS caused by a novel mutation in *INSR*. The trigger for the diagnosis of the 17-year-old boy in the family was a urinalysis at school. Genetic testing showed that his mother and grandfather had the same mutation, but their clinical courses were different.

## Case presentation

A 17-year-old boy was referred to our endocrinology clinic for investigation of hyperinsulinemia. Glycosuria had been noted during a health checkup at high school, and the boy was found to have extreme hyperinsulinemia with a normal glucose concentration. His height, body weight, and body mass index were 165 cm, 52.6 kg, and 19.3 kg/m^2^, respectively (Table 1). He was healthy. He had no outward features of hyperinsulinemia, including acanthosis nigricans or hirsutism, a normal birth weight of 3000 g, and no mental retardation. He had a family history of diabetes in relatives on his mother’s side.

**Table 1:** Clinical characteristics of the family.

Characteristic	Proband	Mother	Grandfather
Age (years)	17	50	87
Height (cm)	165	156	165
Body weight (kg)	52.6	56	45.3
Body mass index (kg/m^2^)	19.3	23	16.6
FPG (mg/dL)	75	107	117
Insulin (μU/mL)	50	37	125.3*
C-peptide (ng/mL)	2.08	2.62	7.6
Proinsulin (pmol/L)	5.4	ND	ND
HbA1c (%)	5.6	6.5	6.4
Triglycerides (mg/dL)	87	114	109
HDL cholesterol (mg/dL)	70	72	43
LDL cholesterol (mg/dL)	82	125	96
Anti-IR antibody	None	ND	ND
Birth weight (g)	3000	3900	Unknown
Acanthosis nigricans	None	None	None
Hirsutism	None	None	None
Diabetes treatment	None	None	DPP4I
Estimated diabetes onset	No diabetes	50 years	77 years

*Postprandial value.

Anti-IR, anti-insulin receptor; BMI, body mass index; DPP4I, dipeptidyl peptidase 4 inhibitor; FPG, fasting plasma glucose; HbA1c, hemoglobin A1c; HDL, high-density lipoprotein; LDL, low-density lipoprotein; ND, not determined.

## Investigation

The patient’s fasting glucose concentration was 75 mg/dL and his hemoglobin A1c (HbA1c) level was 5.6%. Plasma concentrations of insulin, C-peptide, and proinsulin were 50 μU/mL, 2.08 ng/mL, and 5.4 pmol/L, respectively (Table 1). The molar ratios of insulin–C peptide and proinsulin–insulin were 0.44 (normal: 0.1–0.2) and 0.02 (normal: 0.04–0.23), respectively. An oral glucose tolerance test (OGTT) was performed (Table 2). Plasma glucose concentrations were in the normal range. However, insulin concentrations were considerably elevated (0 min: 71 μU/mL; 60 min: 953 μU/mL), suggesting severe insulin resistance. We performed the insulin tolerance test (Table 3). Blood samples were collected before and 3, 6, 9, 12, and 15 min after intravenous injection of regular insulin (0.1 U/kg). The plasma glucose disappearance rate (KITT) was calculated from the linear slope of the plasma glucose concentration curve between 3 and 15 min, as described previously (4). KITT has been reported to be strongly correlated with the glucose infusion rate in a glucose clamp study and has been used for the assessment of insulin resistance (4, 5). The calculated KITT value was 1.91%/min, which was considered to be insulin resistance (mean values of normal, obese, and diabetic subjects are 5.65 ± 0.35, 4.14 ± 0.52, and 2.73 ± 0.29, respectively) (4). Anti-insulin receptor antibody was negative. There was no apparent hormonal or metabolic cause, including obesity.

**Table 2:** OGTT results of the family members.

	Time (min)				
	0	30	60	90	120
Glucose (mg/dL)					
Proband	77	198	193	123	125
Mother	107	221	253	ND	281
Father	98	155	138	ND	91
Brother	94	174	195	143	119
Insulin (μU/mL)					
Proband	71	664	953	840	708
Mother	37	122	188	ND	349
Father	2.6	42.5	30	ND	8.1
Brother	5.4	36.9	78.6	45.2	47.5

ND, not determined; OGTT, oral glucose tolerance test.

**Table 3:** ITT results of the proband.

	Time (min)					
	0	3	6	9	12	15
Glucose (mg/dL)	74	73	74	73	69	63
KITT (%/min)		1.91				

ITT, insulin tolerance test; KITT, plasma glucose disappearance rate.

**Figure 1:**
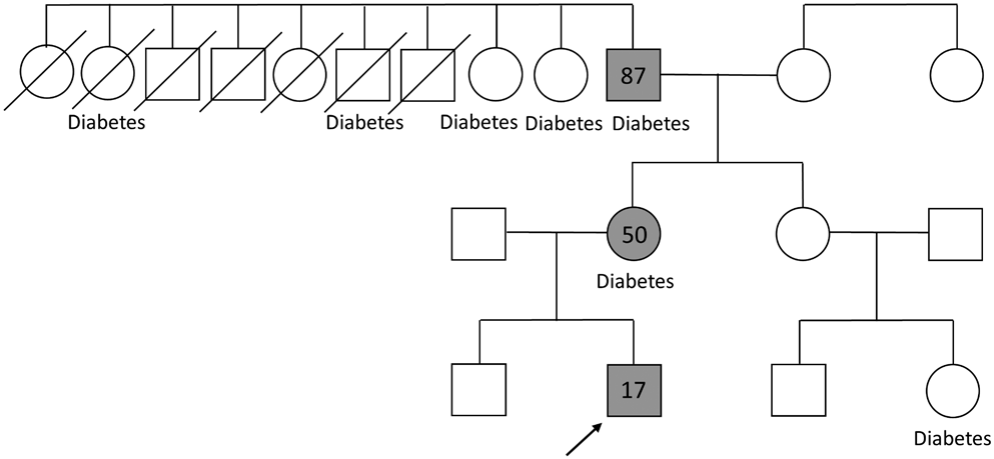
Pedigree of the family. The arrow shows the proband. The gray square and gray circle indicate family members found to have the mutation, and the numbers in the figure represent age in years.

These findings suggested a genetic cause of insulin resistance (6). Therefore, an OGTT was also performed on the patient’s parents and brother (Table 2). His father and brother had normal glucose tolerance and insulin concentrations. However, his mother reached the diagnostic criteria for diabetes and had high insulin concentrations. Her HbA1c level was 6.5%. These findings suggested that the patient had inherited a genetic cause of insulin resistance from his mother. Consistent with this possibility, four of nine of his maternal grandfather’s siblings and his grandfather had diabetes (Fig. 1). The patient’s grandfather was 87 years old. His diabetes was being treated at another clinic by using a dipeptidyl peptidase 4 inhibitor. We found that he also had hyperinsulinemia, with a fasting glucose concentration of 230 mg/dL and a postprandial insulin level of 125 μU/mL. The clinical characteristics of the patient’s mother and grandfather are shown in Table 1. After analysis of these data, type A IRS was highly suspected.

**Figure 2:**
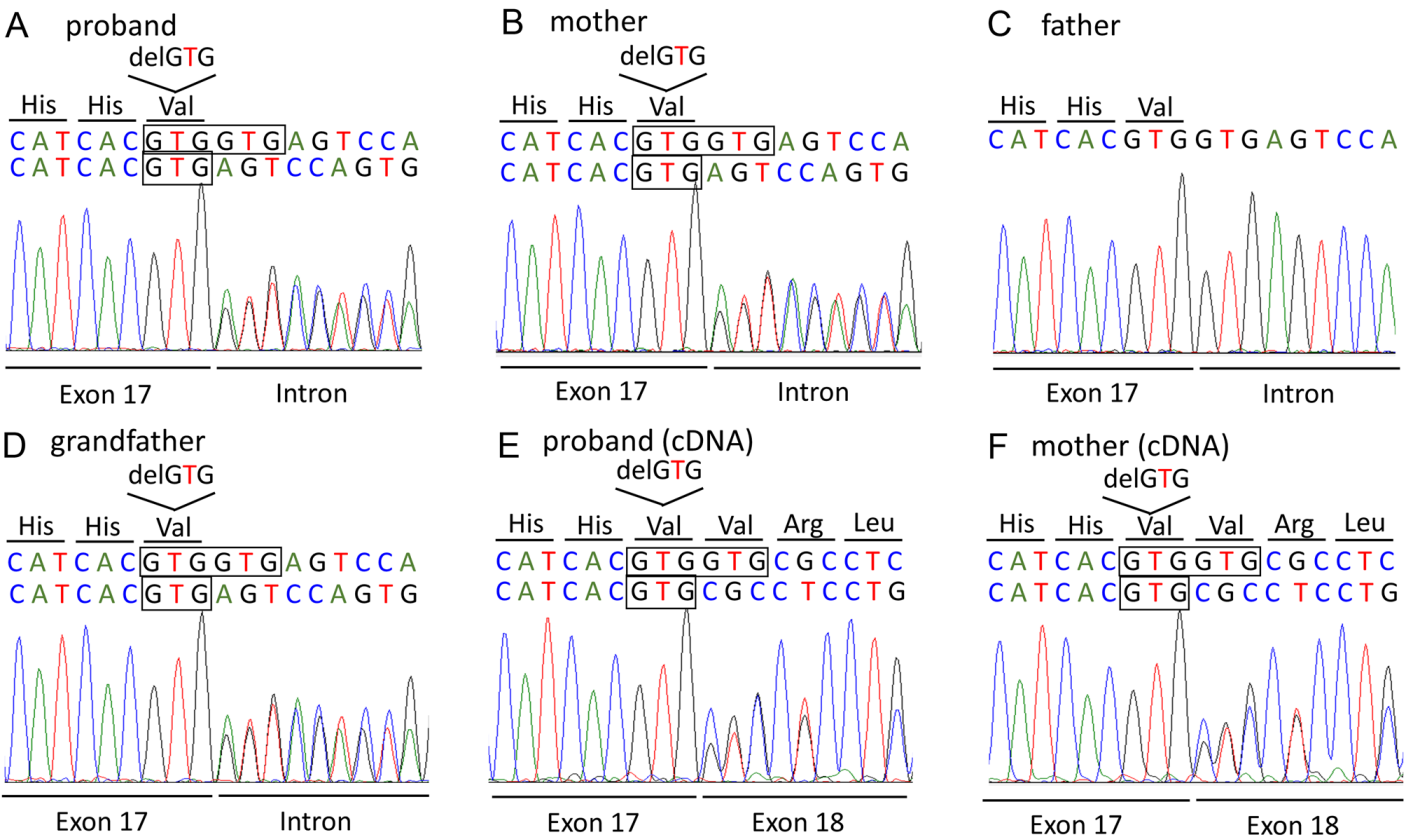
Sequencing results of genomic DNA (A–D) and cDNA (E, F) of the insulin receptor gene (*INSR*). The proband, his mother, and his grandfather have a heterozygous GTG deletion at the 3′ end of exon 17. Sequencing of genomic DNA could not distinguish between this mutation and a GTG deletion next to it at the 5′ end of the intron. Sequencing of cDNA confirmed the mutation as p.Val1086del in exon 17.

We received the consent of the patient (proband), his parents, and his grandfather to perform genetic testing. This research was approved by the ethics committee of the Kobe Graduate School of Medicine (approval no. 170105). We sequenced all 22 exons of the insulin receptor gene (*INSR*, Gene Bank NM_000208.4). Sequencing of the genomic DNA of the patient showed a heterozygous deletion mutation in the 3′ end of exon 17. The specific mutation was c.3256_3258delGTG, p.Val1086del or a deletion of three base pairs in the intron just following them, c.3256+1‗3258+3delGTG (Fig. 2A). The same mutation was identified in the patient’s mother and grandfather, but not in his father who did not have hyperinsulinemia. (Fig. 2A, C and D). We could not distinguish whether the mutation was c.3256_3258delGTG or dc.3256+1‗3258+3delGTG because both are GTG mutations. Therefore, we sequenced the cDNA of the patient and his mother and identified the mutation as p.Val1086del in exon 17 (Fig. 2E and F), which is a novel *INSR* mutation.

## Treatment

The patient did not have diabetes when the genetic test results were confirmed. We planned to follow up twice a year. We advised the patient to maintain a healthy lifestyle and a body mass index within the normal weight range. Six months later, the HbA1c level of the patient’s mother was elevated (6.8%), and we started to administer 250 mg of metformin daily.

## Outcome and follow-up

The HbA1c level of the patient was not elevated 2 years later. Treatment of the patient’s mother with 250 mg of metformin daily was not effective (7.0% at 6 months after starting treatment) and was increased to 500 mg daily. The HbA1c level dropped to 6.5% at 3 months after this increase. The HbA1c level of the grandfather was stable, and information on his clinical course to date was sought to predict the clinical course of his daughter and grandson (the proband). We found that his HbA1c level had been 5.1, 6.6, and 6.9% at 67, 77, and 87 years, respectively. He had begun taking a dipeptidyl peptidase 4 inhibitor at 77 years of age and was still being treated with the same regimen. We estimated that his onset of diabetes was at 77 years of age (Table 1). His HbA1c levels have never exceeded 7.1%.

## Discussion

We describe a family with type A IRS. A 17-year-old boy (the proband) presented with severe insulin resistance possibly caused by a novel deletion mutation of the B subunit of *INSR*. The trigger for the diagnosis of the proband was glycosuria, which was found at a health checkup at high school. The patient’s mother and grandfather had the same genetic mutation, and we studied this family (spanning three generations) to better understand the natural history of this syndrome.

Mutations of the *INSR* gene result in extreme insulin resistance and dysglycemia (7) because of the dysfunction of INSR. Disorders associated with mutations of *INSR* have various phenotypes. Rabson–Mendenhall syndrome (RMS) and Donohue syndrome have the most severe insulin resistance, whereas type A IRS displays milder manifestations (1, 8). Homozygous or compound heterozygous mutations are thought to cause severe syndromes (RMS and Donohue syndrome) (9). Heterozygous mutations cause insulin resistance by a dominant negative effect but should lead to the formation of some fully functional wt/wt receptors, which would result in a less severe phenotype (9).

Mutations causing type A IRS rather than RMS or Donohue syndrome are more frequently found in the tyrosine kinase (TK) domain (9). Some mutations in the TK domain result in decreased tyrosine activity of INSR (10, 11). The deletion mutation Val1086del found in the family of our patient was a novel mutation. Although we cannot conclude that this mutation was definitely the cause of the insulin resistance in this family, Val1086 is in a TK domain near the cluster of tyrosine phosphorylation sites at positions 1158, 1162, and 1163. Therefore, this deletion mutation may lead to decreased TK activity of INSR. However, a functional analysis is required to prove how the mutation affects the function of INSR.

Type A IRS is considered to be rare. However, a nationwide survey conducted in Japan from 2014 to 2016 identified 23 cases of type A IRS and 10 cases of RMS/Donohue syndrome (3). On the basis of a genetic analysis of these cases using the Hardy–Weinberg principle, we estimate that at least 0.05% of the population might harbor a pathological *INSR* mutation in one allele (3). Owing to the mild phenotype of type A IRS and a lack of understanding of this disorder, many of these patients may be misdiagnosed and incorrectly treated. According to a nationwide survey in Japan, 39% of individuals with type A IRS were identified by urinalysis at school, and it was the largest trigger for diagnosis (3). New classification and diagnostic criteria for IRS have been recently published on the basis of the results of a nationwide survey conducted in Japan (6). According to this survey, the major feature of IRS is the presence of hyperinsulinemia (fasting serum insulin concentration of >30 μU/mL) with no apparent cause of insulin resistance, such as obesity or other conditions. Our patient and his mother met those criteria. Clinicians and members of the public need to be aware of the possibility of such genetic syndromes of insulin resistance.

We examined the phenotypes of the patient’s mother and grandfather to gain an understanding of the natural course of type A IRS. The OGTT of the proband’s mother showed hyperinsulinemia, but it was attenuated compared with that of her son (Table 2). The continuation of hyperinsulinemia leads to the failure of β-cell function, which suggests that decreased insulin secretion in the patient’s mother resulted in an abnormal HbA1c level of 6.5%. To support this conclusion, some patients with *INSR* variants require high-dose insulin therapy in the later stages of the disease process (12). Another patient showed a tendency for a progressive increase in post-load glucose concentrations and a decrease in insulin concentrations during an OGTT over 8 years (13).

The onset of diabetes in the proband’s grandfather was estimated to be 77 years, and this is a later onset of diabetes than that in his daughter. Several reports showed that the phenotype, such as the severity of diabetes or insulin resistance with or without acanthosis nigricans, was different within the family, despite them having the same mutation (10, 14, 15). The exact reasons for this finding are unknown. Other genetic and/or environmental factors, such as age and sex, may contribute to the different phenotypes. Additionally, an unknown abnormality in the insulin signaling pathway might have been inherited from the patient’s grandmother. However, we speculate that changes in Japanese lifestyles over the past century affected the diabetes onset time in these two individuals. At the time of writing, the proband does not have diabetes, but he may develop diabetes earlier than his mother because lifestyles are still changing in Japan. Therefore, follow-up is important so that treatment can be started promptly. Moreover, maintaining a healthy lifestyle and a body mass index within the normal weight range is important. The patient’s mother and grandfather have the same mutation, but their diabetes onset times were different, suggesting that lifestyle factors can affect the clinical course.

We chose metformin for the treatment of the proband’s mother. This choice was made because the inhibition of hepatic gluconeogenesis by insulin-independent multiple molecular mechanisms is thought to be largely responsible for the glucose-lowering effect of metformin (16). Metformin was the most prescribed medication in a nationwide survey in Japan (3). In this previous study, among 23 cases of identified type A IRS, 13 (57%) patients were treated with metformin, followed by insulin in 8 (35%) patients. In fact, the effectiveness of metformin for the treatment of type A IRS has been reported (17, 18). However, no established treatment strategy, including metformin, has been established for type A IRS. Long-term observation of the patient’s mother needs to be performed.

In conclusion, we describe a family with type A IRS possibly caused by a novel mutation in the B subunit of INSR. The prevalence of type A IRS might be more frequent than previously thought. Therefore, we need to be aware of the possibility of such genetic syndromes of insulin resistance. This case report suggests that the clinical course of type A IRS can be affected by lifestyle factors.

## Declaration of interest

The authors declare that there is no conflict of interest that could be perceived as prejudicing the impartiality of this case report.

## Funding

This study did not receive any specific grant from any funding agency in the public, commercial or not-for-profit sector.

## Patient consent

Written informed consent for publication of their clinical details was obtained from the patient, his parents and his grandfather for publication of the case report.

## Author contribution statement

OH and SU prepared the manuscript. OH, MY, DS and YF were endocrinologists involved in the clinical care of the patient. YO was an endocrinologist involved in the clinical care of the grandfather. FT was the endocrinologist who identified the hyperinsulinemia of the proband. TT and YH performed the genetic analysis. YH, Professor HM and Professor WO critically reviewed the manuscript. All authors agree to be accountable for all aspects of the work and each contributed considerably to the work.
